# Current distribution of the invasive mosquito species, *Aedes koreicus* [*Hulecoeteomyia koreica*] in northern Italy

**DOI:** 10.1186/s13071-015-1208-4

**Published:** 2015-12-01

**Authors:** Fabrizio Montarsi, Andrea Drago, Simone Martini, Mattia Calzolari, Francesco De Filippo, Alessandro Bianchi, Matteo Mazzucato, Silvia Ciocchetta, Daniele Arnoldi, Frédéric Baldacchino, Annapaola Rizzoli, Gioia Capelli

**Affiliations:** Istituto Zooprofilattico Sperimentale delle Venezie, Legnaro, Italy; Entostudio s.r.l, Brugine, Italy; Istituto Zooprofilattico Sperimentale della Lombardia e dell’Emilia Romagna, Brescia, Italy; Mosquito Control Laboratory, QIMR Berghofer Medical Research Institute, Brisbane, Australia; Queensland University of Technology, Brisbane, Australia; Department of Biodiversity and Molecular Ecology, Research and Innovation Centre, Fondazione Edmund Mach (FEM), San Michele all’Adige, Italy

**Keywords:** *Aedes koreicus*, Invasive mosquito species, Entomological surveillance, Italy

## Abstract

**Background:**

The invasive species *Aedes* (*Finlaya*) *koreicus* was first identified in north-eastern Italy in 2011, during the ongoing surveillance activity of *Aedes albopictus*. Following this finding, a more intensive monitoring was carried out to assess the distribution of the species and to collect biological data. Herein, we report the new records obtained by four years of surveillance.

**Findings:**

The presence of *Ae. koreicus* was checked using ovitraps, adults traps and by larval collections in all possible breeding sites from May 2011 to July 2015. The monitoring started in the site of the first detection (Province of Belluno) and was then extended in the neighbouring Provinces belonging to four Regions. *Aedes koreicus* was found in 73 municipalities out of 155 monitored (47.1 %), including 23 municipalities (14.8 %) previously not infested. The area of first detection of *Ae. koreicus* (Province of Belluno) was also the most infested (68 %). However the mosquito has also been found to the west (Province of Trento) and to the south and south-west (Provinces of Vicenza and Treviso) of the initially infested area.

**Conclusions:**

The spread of *Ae. koreicus* is directed towards south and west from the original infested area, likely due to the dense road connections and the habitat suitability of the new areas. According to these records, northern Italy has a high probability to be invaded by *Ae. koreicus* in the next decade. These data can be useful to validate predictive models of potential distribution and dispersal of this species in Italy or in Europe.

**Electronic supplementary material:**

The online version of this article (doi:10.1186/s13071-015-1208-4) contains supplementary material, which is available to authorized users.

## Findings

Since the discovery of the invasive mosquito species (IMS) *Aedes albopictus* (Skuse) or Asian tiger mosquito in north-eastern Italy in 1991 [1], many local surveillance and control programs were started. The entomological survey is primarily based on the use of ovitraps in areas where the tiger mosquito is present, while in non-colonized areas, collection of larvae/pupae and adult trapping are the best methods for its early detection. In 2011, during the surveillance activity in a tiger mosquito-free area of the Veneto Region, an unexpected mosquito was caught with different features from *Ae. albopictus*. Larvae and adults were then morphologically and molecularly identified as *Aedes* (*Finlaya*) *koreicus* (Edwards) [2]. The finding of *Ae. koreicus* in Italy represents the second incursion in Europe [2], after a previous report in Belgium in 2008 [3]. After the first Italian record, a more intensive monitoring was carried out to follow the distribution of *Ae. koreicus* and to collect biological data on its life cycle.

In this work we update the results of four years of surveillance on the presence and spread of *Ae. koreicus* in Italy. The survey started in 2011 from Valbelluna (Province of Belluno, northern Italy), where the first *Ae. koreicus* mosquitoes were found. A previous study reported that this species was well established in an area of 2600 km^2^ [4]. Since 2012, the monitoring has been extended in the whole Province of Belluno and in the neighbouring Provinces (Vicenza, Treviso, Trento, Pordenone and Verona). An additional monitoring was carried out in north-western Italy (Lombardia Region) in 2014, due to its proximity to another infested area in Switzerland [5]. All possible breeding sites, such as catch basins, man-made containers, tires and natural mosquito larval habitats were checked and the larvae collected. The places visited included private and public places (private gardens, garden centres and florists, tire markets, cemeteries, farms and houses). In addition, standard ovitraps and adults traps (BG-Sentinel™ traps baited with CO_2_ and lure) were used in some Provinces (Trento, Belluno, Vicenza and Sondrio). The collection sites were georeferenced. Mosquitoes were sampled from public areas according to the official guidelines of the Regional Health Authorities. Mosquitoes sampled from private areas were collected after verbal consensus of the owners.

The specimens were morphologically identified [6, 7]. In case of doubtful morphological identification or findings in new areas, a molecular confirmation was carried out using a PCR [8].

**Fig. 1:**
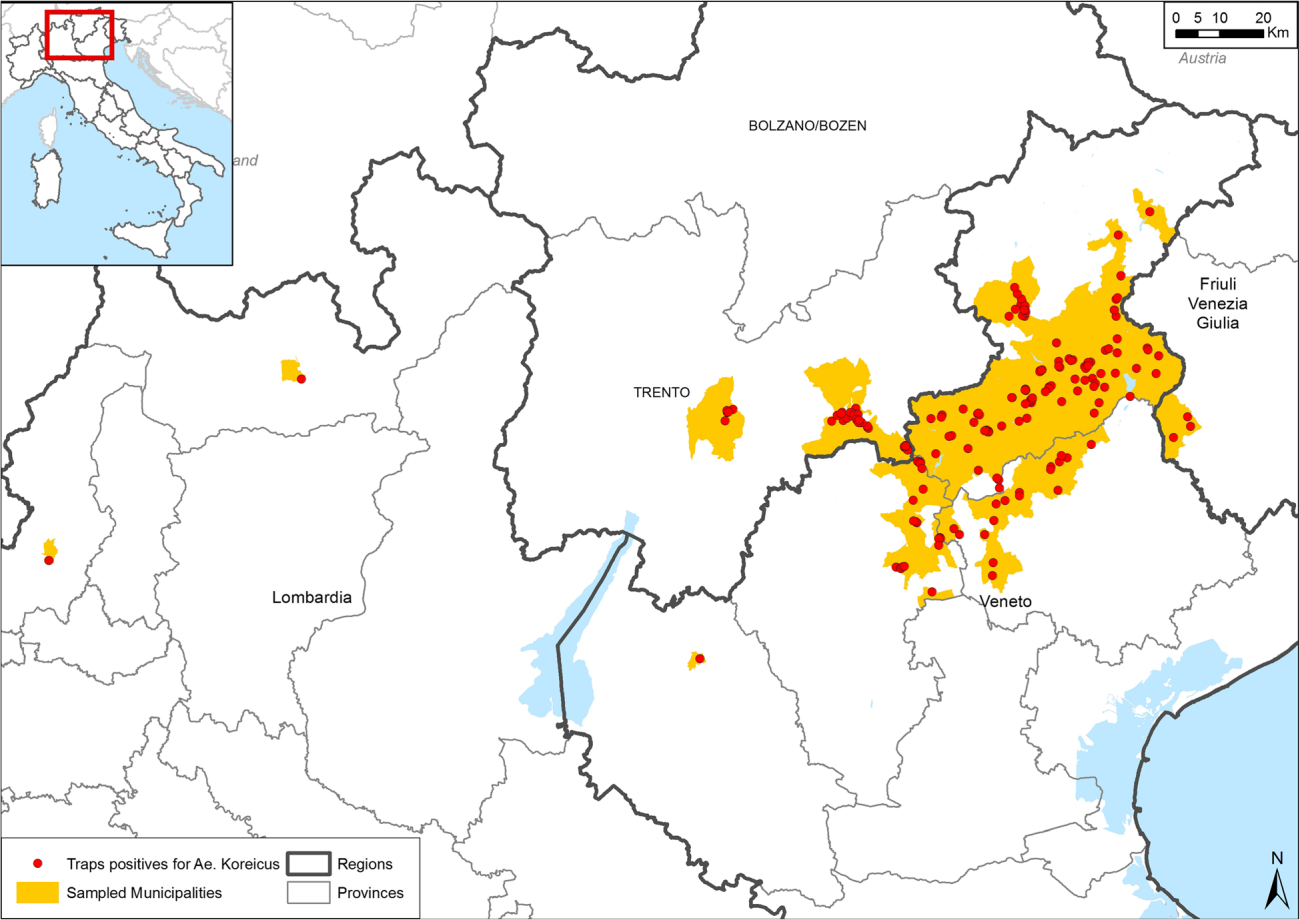
Map of the municipalities monitored and positive sampling sites for the presence of *Aedes koreicus* in northern Italy, 2011–2015

The current distribution (July 2015) of *Ae. koreicus* in northern Italy is reported in Fig. 1.

*Aedes koreicus* was found in 73 municipalities out of 155 monitored (47.1 %) located in four Regions (Table 1). The mosquito was found in 23 municipalities (14.8 %) that were negative in 2011. Larvae were found mainly in artificial containers close to houses, in gardens and in places with stored materials. Compared to *Ae. albopictus*, *Ae. koreicus* was less present in catch basins and in areas totally urbanized (city/town centre). *Aedes koreicus* was found mainly in hilly and mountainous areas in a range of altitude between 42 m.a.s.l. (Polcenigo, Province of Pordenone, Lat 45°43′30.5″N; Long 11°54′37.7″E) and 1250 m.a.s.l. (Pedavena, Province of Belluno: Lat 46°04′12''; Long 11°50′31'').

**Table 1:** Municipalities monitored and positive for *Aedes koreicus* in northern Italy, 2011–2015

Region	Province	Municipalities monitored/present (%)	Municipalities positive/monitored for *Ae. koreicus* (%)
Veneto	Belluno	50/67 (75.4 %)	34/50 (68.0 %)
	Treviso	26/95 (23.2 %)	14/26 (53.8 %)
	Vicenza	15/121 (12.4 %)	8/15 (53.3 %)
	Verona	4/98 (4.1 %)	1/4 (25.0 %)
Trentino Alto Adige	Trento	42/217 (19.3 %)	11/42 (26.2 %)
Friuli Venezia Giulia	Pordenone	8/50 (16.0 %)	3/8 (37.5 %)
Lombardia	Sondrio	1/78 (1.3 %)	1/1 (100.0 %)
	Brescia	4/206 (1.9 %)	0/4 (0.0 %)
	Bergamo	2/242 (0.8 %)	0/2 (0.0 %)
	Lecco	1/88 (1.1 %)	0/1 (0.0 %)
	Como	2/154 (0.6 %)	1/2 (50.0 %)
Total		155/1416 (10.9 %)	73/155 (47.1 %)

The location of the first detection in Italy (Province of Belluno) was also the most infested.

Several areas where *Ae.koreicus* is now present were not infested before 2012, such as seven municipalities in province of Belluno, three in province of Trento (west of initially infested part of Belluno) and six both in province of Vicenza and Treviso (south-west and south of initially infested part of Belluno). Moreover, unexpected new records scattered in a wide area of northern Italy were found in the last two years (Tavernerio and Sondrio, Lombardia Region; Cerro Veronese, Veneto Region, Caneva and Polcenigo, Friuli Venezia Giulia Region). The spread of *Ae. koreicus* seems to be faster towards south and west from its original and more infested area (Province of Belluno), likely due to the dense road connections and the habitat suitability of new areas. On the contrary, the distribution of *Ae. koreicus* in Belgium is still limited in a small area of 6 km^2^ [3].

It is not possible to track back the route of entry and the time of arrival of *Ae. koreicus* in this part of Italy. This species probably arrived in the province of Belluno where it was found for the first time and where the current density of population is the highest in the country. During the last years, the new records of *Ae. koreicus* in Italy could originate either from new introductions from its native range or dispersion from colonized areas, supported by human activities through private vehicles, public transport and/or freight. Previous introductions of IMS in USA and Europe suggest that the trade of used tires and plants is the main cause of invasion in a new area [9]. Through this way, new introductions of invasive *Aedes* species can be frequent as reported in The Netherlands [10].

There is little information on the factors that are driving the dispersal of *Ae. koreicus*. The probability of establishment and spread of IMS depends on environmental parameters, host availability and suitable climatic conditions [11]. The knowledge of these features is of particular importance to predict its future spread. Probably, the climate is not the only factor limiting the distribution in Europe since Korea, Belgium and northern Italy have similar climates [12].

*Aedes koreicus* is native to Korea, Japan, China and eastern Russia. Little information is available on this species, but recent studies are clarifying some aspect of its biology and ecology in the new-colonized areas [3, 4, 13]. *Aedes koreicus* is reported to feed on humans and domestic animals, and it seems to be well adapted to urban environment [13, 14]. Like other species belonging to genus *Aedes*, *Ae. koreicus* females lay eggs that can survive during the winter and hatch in the spring [15, 16]. In comparison with the Asian tiger mosquito, this species seems to be better adapted to colder temperature [16], a feature that likely favoured its establishment and survival in hilly and pre-alpine areas of Italy.

**Fig. 2:**
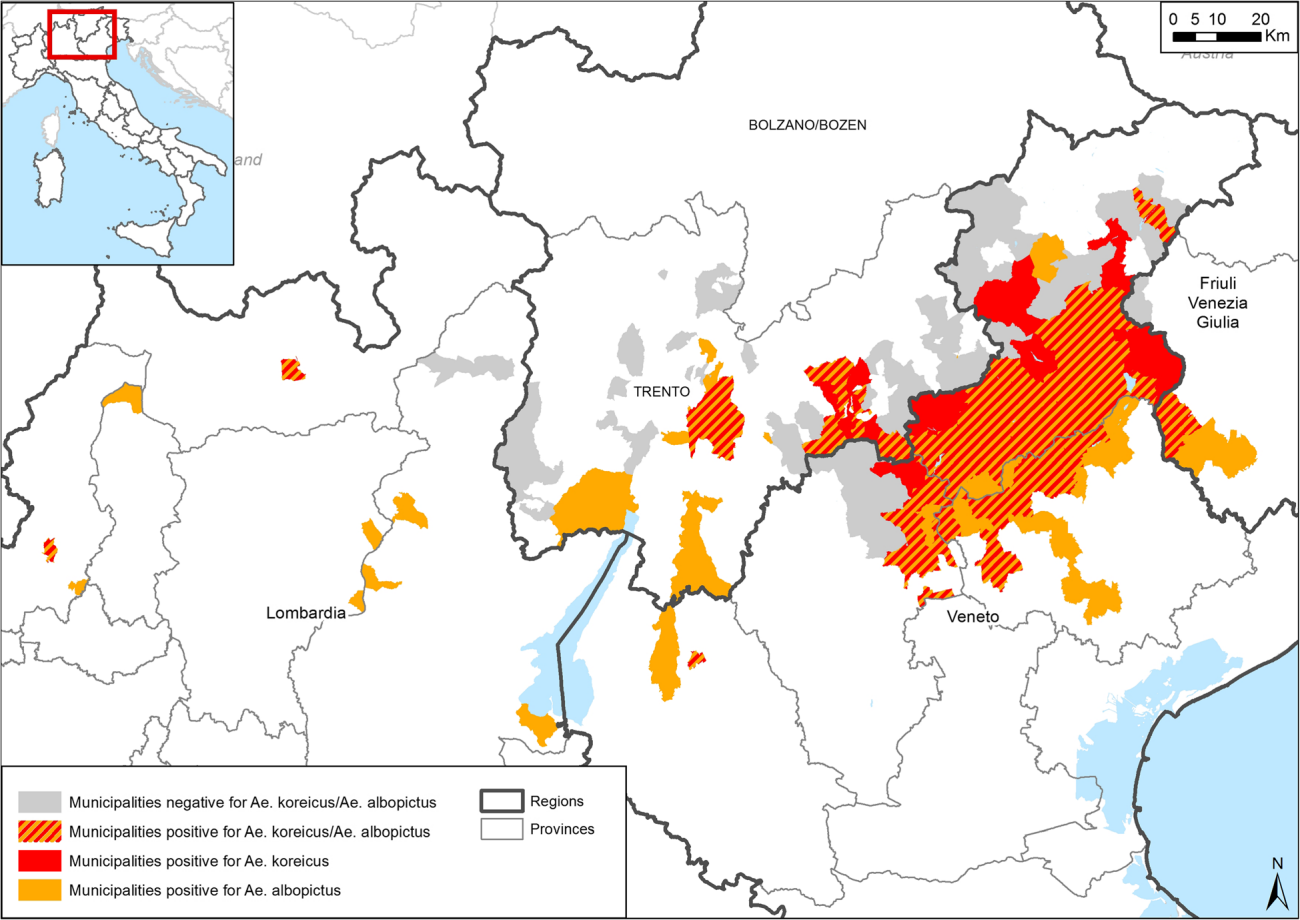
Map of municipalities positive for the presence of *Aedes koreicus*, *Aedes albopictus* and their overlapping areas in northern Italy, 2011–2015. The names of municipalities are available in the Additional file 1

The results of our monitoring shows a large area of *Ae. koreicus* and *Ae. albopictus* co-habitation (Fig. 2; Additional file 1). Interspecific competition between *Ae. koreicus* and *Ae. albopictus* might occur in many areas with mild climate conditions as reported in other countries among invasive and native container-breeding mosquitoes [17]. Preliminary results of larval competition experiments in laboratory suggested that the development of *Ae. koreicus* might be negatively affected by the presence of *Ae. albopictus* (Frédéric Baldacchino, personal communication). In nature in our study, *Ae. albopictus* let a spatio-temporal gap to *Ae. koreicus*, avoiding larval competition as observed for *Ae. japonicus* [18]. Indeed, *Ae. koreicus* larvae are found early in the spring (approximately one month before *Ae. albopictus* larvae) and in areas too cold for *Ae. albopictus* where *Ae. koreicus* can occupy an empty niche.

The establishment of *Ae. koreicus* in an area where *Ae. albopictus* is already present has complicated the current entomological monitoring system, because larvae and adults are difficult to recognize and their eggs are practically indistinguishable.

*Aedes koreicus* has been suggested as a potential vector of arboviruses [19]. The species is reported to be involved in the transmission of the Japanese encephalitis virus, however without laboratory confirmation of its vector competence [20]. More evidences show that *Ae. koreicus* can transmit the dog heartworm, *Dirofilaria immitis* [21, 22].

According to these records, northern Italy has a high probability to be invaded by *Ae. koreicus* in the next decade. These data can be useful to validate predictive models of potential distribution and dispersal of this IMS in Italy or in other parts of Europe.

Therefore, early detection of IMS distribution is needed to prevent new establishments and to plan control actions. Unfortunately the eradication of an establish species is very unlikely [23].

## Additional file

Additional file 1:
**List of municipalities currently positive (July 2015) for** ***Aedes koreicus*** **and** ***Aedes albopictus*** **in northern Italy.** (XLS 47 kb)
